# Loneliness among dementia caregivers: evaluation of the psychometric properties and cutoff score of the Three-item UCLA Loneliness Scale

**DOI:** 10.3389/fpsyt.2025.1526569

**Published:** 2025-04-07

**Authors:** Amira Mohammed Ali, Saeed A. Al-Dossary, Carlos Laranjeira, Abeer Selim, Souheil Hallit, Abdulmajeed A. Alkhamees, Aljawharah Fahad Aljubilah, Musheer A. Aljaberi, Ebtesam Abdullah Alzeiby, Annamaria Pakai, Haitham Khatatbeh

**Affiliations:** ^1^ Department of Psychiatric Nursing and Mental Health, Faculty of Nursing, Alexandria University, Alexandria, Egypt; ^2^ Department of Psychology, College of Education, University of Ha’il, Ha’il, Saudi Arabia; ^3^ School of Health Sciences, Polytechnic University of Leiria, Leiria, Portugal; ^4^ Centre for Innovative Care and Health Technology (ciTechCare), Polytechnic University of Leiria, Leiria, Portugal; ^5^ Comprehensive Health Research Centre (CHRC), University of Évora, Évora, Portugal; ^6^ Faculty of Nursing, Psychiatric and Mental Health Nursing Department, Mansoura University, Mansoura, Egypt; ^7^ College of Nursing, King Saud bin Abdulaziz University for Health Sciences, Riyadh, Saudi Arabia; ^8^ King Abdullah International Medical Research Center, Riyadh, Saudi Arabia; ^9^ School of Medicine and Medical Sciences, Holy Spirit University of Kaslik, Jounieh, Lebanon; ^10^ Psychology Department, College of Humanities, Effat University, Jeddah, Saudi Arabia; ^11^ Applied Science Research Center, Applied Science Private University, Amman, Jordan; ^12^ Department of Psychiatry, College of Medicine, Qassim University, Al Qassim, Saudi Arabia; ^13^ Department of Psychology, College of Education and Human Development, Princess Nourah bint Abdulrahman University, Riyadh, Saudi Arabia; ^14^ Department of Internal Medicine, Section Nursing Science, Erasmus University Medical Center (Erasmus MC), Rotterdam, Netherlands; ^15^ Institute of Nursing Sciences, Basic Health Sciences and Health Visiting, Faculty of Health Sciences, University of Pécs, Pécs, Hungary; ^16^ Faculty of Nursing, Yarmouk University, Irbid, Jordan

**Keywords:** Three-item version of the University of California Los Angeles Loneliness Scale (UCLALS3)/loneliness, caregiving burden/burnout/the Zarit Burden Interview (ZBI), psychological distress/depression anxiety stress scale 8-items, factor structure/psychometric, older adults/old age/elders/elderly, informal/family caregivers, cutoff score/receiver-operating characteristic curve (ROC) analysis

## Abstract

**Introduction:**

Dementia is a chronic progressive syndrome, with an entire loss of function in the late stages. The care of this demanding condition is primarily provided by family members, who often suffer from chronic burnout, distress, and loneliness. This instrumental study aimed to examine the factor structure, reliability, convergent validity, criterion validity, and cutoff scores of a short loneliness measure: the Three-Item version of the University of California, Los Angeles, Loneliness Scale (UCLALS3) in a convenience sample of dementia family caregivers (N = 571, mean age = 53 ±12 years, 81.6% females).

**Methods:**

Exploratory and confirmatory factor analyses were used to examine the structure of the UCLALS3 while receiver-operating characteristic (ROC) curve, including caregiving burden and emotional distress as outcomes, was used to examine its cutoff.

**Results:**

One factor accounted for 79.0% of the variance in the UCLALS3; it was perfectly invariant across genders but variant at the metric level across countries. The scale had adequate internal consistency (alpha = 0.87), high item-total correlations (0.69 – 0.79), reduced alpha if item deleted (0.77 – 0.86), and strong positive correlations with caregiving burden and psychological distress scores (r = 0.57 & 0.74, p values = 0.01). Percentile scores and the ROC curve suggested two cutoffs (≥6 and ≥6.5), which classified 59.3 and 59.4% of the participants as having higher levels of loneliness—comparable to global levels of loneliness among informal caregivers. The Mann-Whitney test revealed significantly high levels of caregiving burden and distress in caregivers scoring ≥6.5 on the UCLALS3.

**Conclusion:**

The UCLALS3 is a valid short scale; its cutoff ≥6.5 may flag major clinically relevant symptoms in dementia caregivers, highlighting the need for tailored interventions that boost caregivers’ individual perception of social relationships. More investigations are needed to confirm UCLALS3 invariance across countries.

## 1 Introduction

Loneliness is a feeling of insurmountable distance between self and others (1). Lonely individuals develop unpleasant experiences as a result of deficiencies in their network of social relations, which may be of quantitative (e.g., network size, number of friends) and/or qualitative aspects (e.g., relationship closeness, trust, intimacy) (2, 3). Loneliness has also been classified as social deficit in the amount and quality of relations as well as an emotional deficit in relationship closeness (4). Loneliness represents a core public issue worldwide, with a stable prevalence of 15–30% in the general population (5, 6). However, its prevalence varies over regions of the world and lifetime, with peaks among adolescents (14.4%) and older adults (24.2%) while lower rates are reported in middle adulthood (2, 6). Although loneliness represents a stable trait, the set point for the loneliness feeling vacillates according to specific social circumstances (7).

Loneliness may result from the symptoms and maladaptive behaviors that develop in certain pathological conditions such as withdrawing and not confiding in depressive disorders (1, 8). It also increases in response to situational threats to a cherished interpersonal relationship such as social exclusion, ostracism, rejection, separation, divorce, and bereavement (7). Loneliness has increased in the last few decades due to the spread of activities, which limit the chances of social interactions such as digital gaming as well as excessive use of the internet and smartphones (9–12). Social distancing measures have been extensive during the long-lasting COVID-19 pandemic, which has been associated with accelerated internet use for gaming, and social media interactions, and receiving information on the pandemic, leading to reduced life satisfaction, depressed mood, and increased chance for social connections (10, 13–18). Lonely people may retreat to social media to meet their needs for inclusion in a social group (19, 20). Unfortunately, creating virtual relations through social media does not mitigate loneliness, and it may evoke a range of physical and mental adverse effects such as nomophobia (fear of being disconnected from smartphones), body dissatisfaction, and disordered eating, among others (12, 21). Even more, a recent meta-analysis reports a very low quality of evidence for the effect of digital interventions on loneliness among the elderly (5).

Loneliness at moderate to severe levels is widely reported among dementia family caregivers (up to 61.4) (4, 22–24). The time and effort exerted in caregiving may be a source of distancing and relational deprivation in this population (24). Among dementia caregivers, loneliness is aggravated by increased social isolation and caregiving stress. On the other hand, loneliness levels are lower among those expressing good quality of relationship with the care recipient, as well as those with increased levels of well-being and life satisfaction (4, 24, 25). The adverse effects of loneliness on the health and well-being of caregivers can be devastating. Loss of self-esteem has been reported by dementia spouse caregivers (22, 24). Loneliness may also worsen mood and perpetuate depression (26, 27). Indeed, loneliness was the only predictor of depression among family caregivers (accounting for up to 49% of the variance) (22, 24). Longitudinal data reveals an increase in the physical and mental symptoms (e.g., concurrent pain, depression, and fatigue) and larger increases in symptom cluster levels from one year to the next in lonelier than less lonely caregivers (23). The effects of loneliness, along with the caregiving burden and possible physical and mental problems, which older caregivers (e.g., spouses) may suffer as a function of their advanced age, may put the well-being of those caregivers in jeopardy (14).

Loneliness can be mitigated through a variety of cognitive interventions, which focus on interrupting maladaptive cognitions about the social world (1). Such manipulations may interfere with the psychophysiological mechanisms underlying loneliness-related biopsychosocial morbidity (28). Therefore, it is important to screen for and treat loneliness, particularly in groups vulnerable to stress.

Because the literature is mixed on whether loneliness is a unidimensional or a multidimensional construct, different instruments were designed to measure global loneliness as well as different dimensions of loneliness (3). The University of California, Los Angeles, Loneliness Scale (UCLALS) is a widely used 20-item measure of global loneliness. Although several studies replicated the unidimensional structure of the UCLALS, some studies reported two (29, 30) or three dimensions (31, 32). The scale has been revised several times resulting in many shortened versions, which comprise fewer items ranging from 10 to three (33). The three-item UCLALS3 is widely used because of its brevity; however, it has been tested in a few studies. Until the current moment, no cutoff score has been identified to distinguish victims of loneliness who may require special help. The present study aimed to evaluate the psychometrics of the UCLALS3 in a European sample of dementia family caregivers during the peak of the COVID-19 pandemic. We hypothesized that the UCLALS3 will 1) demonstrate a single factor structure, 2) express adequate internal consistency and convergent validity, and 3) correlate strongly with caregiving burden and psychological distress. A secondary aim of this study was to determine an optimal cutoff score for the UCLALS3.

## 2 Materials and methods

### 2.1 Design, participants and procedure

This instrumental study is a secondary analysis that was based on data obtained from a convenience sample of 571 Italian (74.4%) and Swiss (25.6%) dementia family caregivers (mean age = 53 ± 12, range = 24–89 years) (34). The sample was recruited through an online survey conducted in the Italian language through RedCap over one month from May 25 to June 25, 2020. The study included those aged 18 years or older who could speak Italian and stated being family caregivers of non-institutionalized individuals who are formally diagnosed with dementia. Not consenting to participate or not meeting the inclusion criteria disqualified inclusion in the study. Swiss participants were recruited from a border region in southern Switzerland where more Swiss people can speak Italian (34).

Most participants were females (n = 466, 81.6%); adult children of the dementia patients (n = 410, 71.8%); full or part-time employees (n = 283, 49.6%); and with a higher or secondary education (n = 322, 56.4%). The average duration of dementia caregiving was 6.1 ± 4.0 years. Care recipients were primarily affected with Alzheimer’s type dementia (n = 316, 55.3%) and unable to perform activities of daily living (ADL) (n = 455, 79.7%). The characteristics of the respondents and procedure are described in detail elsewhere (14, 34, 35). The dataset generated and analyzed during the current study is available in the Zenodo open repository, DOI: 10.5281/zenodo.4748651 (36).

### 2.2 Measures

Data were collected through a self-administered online survey, which comprised a set of questions eliciting information on the sociodemographic characteristics of the participants (e.g., age, gender, education, etc.), as well as the condition of dementia care recipients (e.g., dementia type and level of ability to perform ADL independently), duration of care provision, and if they received help with care from someone else. The survey also comprised a test battery, which consisted of three symptom measures:

1. The Italian version of the Three-Item version of the University of California, Los Angeles, Loneliness Scale (UCLALS3) (7, 37). This scale has been developed as a refinement of the 20-item UCLALS for easier screening of loneliness through interviews on the phone. It consists of three items, which measure three interrelated dimensions of isolation, relational connectedness, and trait loneliness: how often do you feel that you lack companionship, how often do you feel left out, and how often do you feel isolated from others. The respondents rate the scale on three response categories (1= hardly ever, 2 = some of the time, and = 3 often). The minimum and maximum total scores of the UCLALS3 range between 3 and 9. Higher scores indicate higher levels of loneliness (7). Its reliability in the current study is very good (see the results section).
2. The Italian version of the Zarit Burden Interview (ZBI) (37) consists of 22 items, which capture burnout: the adverse effects of caring for a patient with dementia on physical and mental health, quality of life, relationships, and finance (38). Example items include feeling strained, feeling angry, social life suffering, financial stress, health affected, and lack of privacy. Respondents rate the frequency of endorsing the items of the ZBI on a five-point Likert scale (0 = never, 1 = rarely, 2 = sometimes, 3 = quite frequently, 4 = nearly always). The response categories of only item 22 are different (0 = not at all, 1 = a little, 2 = moderately, 3 = quite a bit, and 4 = extremely). The minimum and maximum total scores of the ZBI range between 0 and 88. Burnout or higher burden are flagged by higher ZBI scores (37, 39). Its reliability in the current study is excellent (coefficient alpha = 0.94).
3. The Depression Anxiety Stress Scale-8 (DASS-8) was nested within the Italian version of the DASS-21 (40). In other words, the respondents rated the 21 items of the DASS-1, but we used in the analysis only eight items, which comprise the DASS-8. This is because this short version of the DASS-21 demonstrates adequate psychometric qualities relative to the full scale (9, 41). Like the DASS-21, the DASS-8 comprises three subscales, which measure the symptoms of depression, anxiety, and stress, respectively. The scale itself measures overall distress. The depression subscale comprises three items (e.g., felt that I had nothing to look forward), the anxiety subscale also comprises three items (e.g., felt close to panic), while the stress subscale comprises only two items (e.g., was using a lot of my mental energy) (9, 41). The respondents rate the scale on four response categories (0 = did not apply to me at all, 1 = applied to me to some degree, or some of the time, 2 = applied to me to a considerable degree or a good part of the time, and 3 = applied to me very much or most of the time). Accordingly, the minimum score of the DASS-8 and its subscales is 0 while their maximum scores are 24, 9, 9, and 6, respectively. Its reliability in the current study is excellent (coefficient alpha = 0.93).

### 2.3 Statistical analysis

Mean and standard deviation (SD) were used to describe continuous variables while frequency and percentage were used to describe categorical variables. To examine the factor structure of the UCLALS3, we conducted exploratory factor analysis (EFA) with maximum-likelihood extraction, direct Oblimin rotation, Kaiser-Meyer-Olkin (KMO), and Bartlett’s test of sphericity. EFA permits the items of a measure to load freely on the corresponding factors without enforcing any constraints.

In a next stage, confirmatory factor analysis (CFA) was conducted to examine the theory-based structure of the UCLALS3. Because of its limited number of items, examination of the UCLALS3 through CFA produces a saturated model (χ^2^ = 0, DF = 0), which has no explanatory power because the absence of degrees of freedom denotes equity of the number of estimated parameters to the number of data points (42). To escape this problem, CFA is conducted by anchoring the ultra-short scale to another measure in a structural equation model (SEM) (27). Therefore, the UCLALS3 was anchored to the DASS-8. In addition to non-significant chi square index (χ^2^), two pairs of indices were considered as criteria for judging model fit as acceptable or good: 1) Comparative Fit Index (CFI) and Tucker–Lewis Index (TLI) >0.90 or >0.95, and 2) root mean square error of approximation (RMSEA) and standardized root-mean-square residual (SRMR) <0.08 or <0.06, respectively (43, 44).

Measurement invariance of the UCLALS3 across gender and country was evaluated through multi-group analysis, which evaluated four nested models that address configural, metric, scalar, and strict invariance. Because χ^2^ is sensitive to sample size, variant models were determined based on ΔCFI and ΔTLI exceeding 0.020, along with ΔRMSEA above 0.015 (35, 45).

To identify the internal consistency of the UCLALS3, alpha coefficient was estimated. Convergent validity testing was mirrored by item-total correlations and alpha if an item was deleted. The criterion validity of the UCLALS3 was evaluated through Pearson correlations with the scores of the ZBI and the DASS-8.

To identify a suitable cutoff point for the UCLALS3, we examined its percentile scores. We also used the receiver-operating characteristic (ROC) method. In this technique, the UCLALS3 was used as a continuous variable to differentiate caregivers with remarkable caregiving burden based on a ZBI cutoff score of 48 (46), as well as those with a significant level of distress based on a DASS-8 score of 13 (35, 47). The effectiveness of the UCLALS3 as a diagnostic marker can be considered according to the value of the Area Under Curve (AUC), sensitivity, specificity, and the Youden index. The latter is calculated as the sum of sensitivity and specificity minus one. In ideal tests, the values of all these fit indices should be close to 1 (48, 49).

The Mann Whitney U test and Kruskal Wallis test were used to examine differences in the levels of caregiving burden and distress across the examined levels of loneliness as determined by the percentiles and the ROC analysis. The analyses were conducted in SPSS (version 28), and the significance of the results was considered at a probability level less than 0.05 in two-tailed tests.

### 2.4 Ethics

Electronic consent was obtained from all subjects involved in the study. Before answering the main questionnaire, all participants provided informed consent for participation, data collection, and analysis by clicking the “Yes, I agree and give my informed consent” box on the digital form. Responses to the electronic questionnaire were anonymous; no personal data were requested to identify the participant. The current research followed the ethical guidelines of the Helsinki Declaration, ensuring that all participants received the same information while completing the questionnaire. The research was in accordance with the European Data Protection Law (34).

## 3 Results

**Figure 1:**
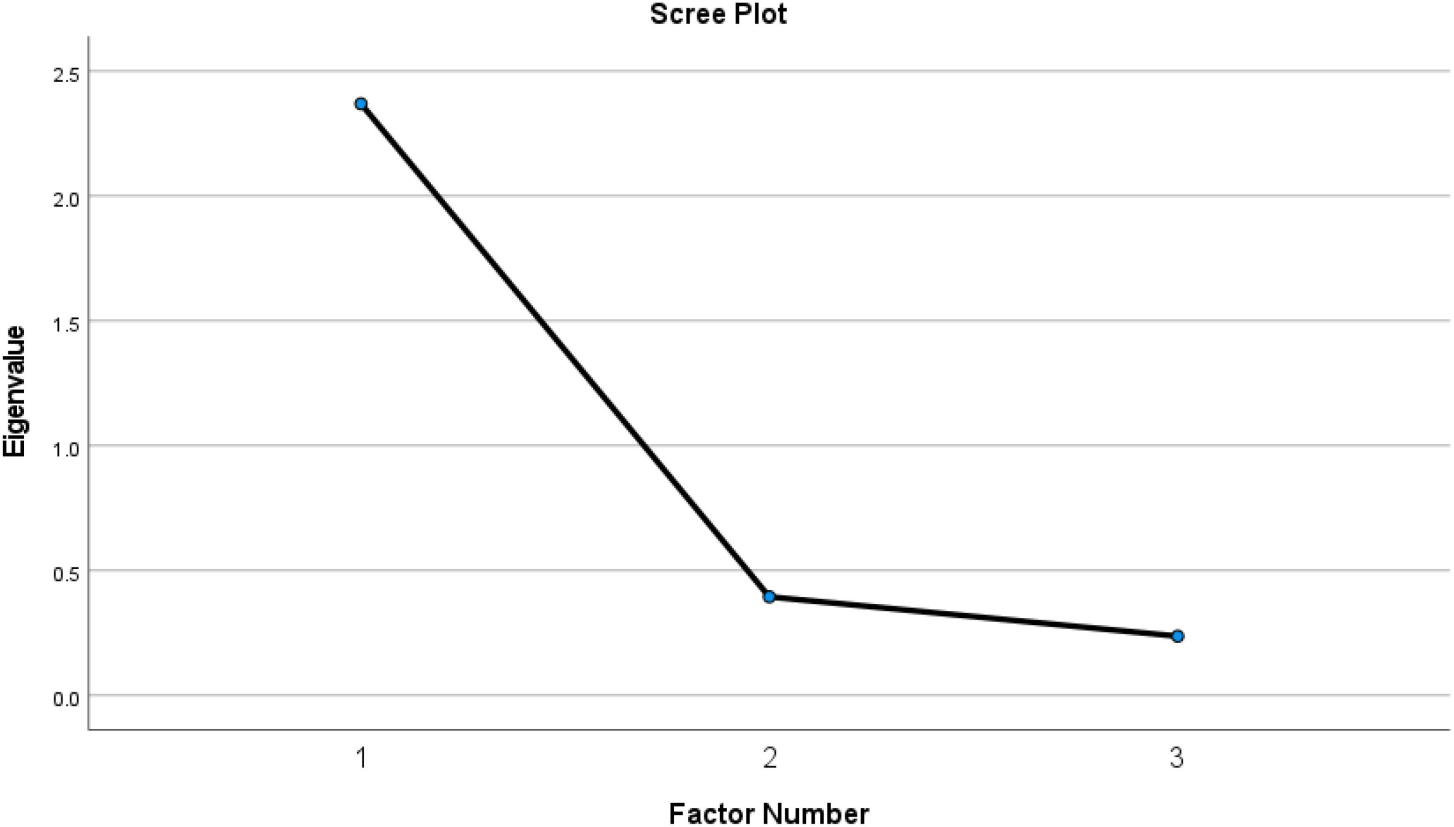
Factor structure of the Three-Item University of California, Los Angeles, Loneliness Scale-version (UCLALS3) among dementia caregivers as determined by exploratory factor analysis.

The values of KMO = 0.721 and Bartlett’s test (χ^2^(3) = 857.50, p < 0.001) indicate adequate sample size and participant-to-item ratio for the EFA. A single factor with an eigenvalue greater than one (2.37) was produced (**Figure 1**). It explained 78.97% of the variance in the UCLALS3. The loadings of all three items on this factor were strong, as shown in **Table 1**.

**Table 1:** Descriptive statistics, item loadings, and internal consistency indicators of the Three-Item University of California, Los Angeles, Loneliness Scale-version (UCLALS3) among dementia caregivers (N = 571).

	Mean	SD	Item loadings from EFA	Alpha if item deleted	Item total correlations
UCLALS -1	2.37	0.72	0.74	0.865	0.687
UCLALS -2	2.16	0.81	0.89	0.773	0.788
UCLALS -3	2.24	0.79	0.86	0.790	0.769

EFA, exploratory factor analysis.

**Figure 2:**
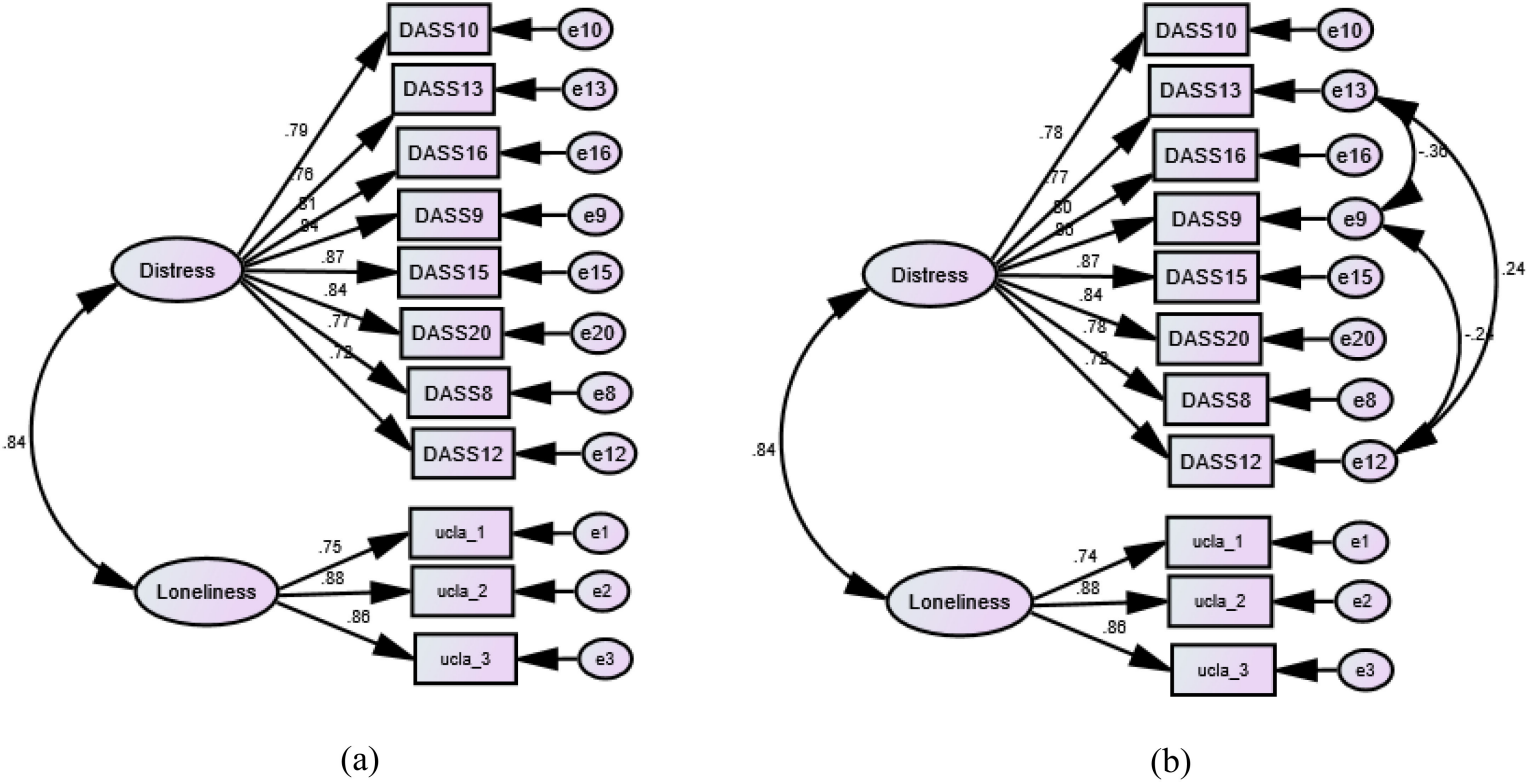
Factor structure of the Three-Item University of California, Los Angeles, Loneliness Scale-version (UCLALS3) among dementia caregivers as determined by confirmatory factor analysis. **(a)** Crude model. **(b)** Modified model with error correlations.

The fit of the SEM was acceptable (χ^2^ (DF) = 255.33 (43), p = 0.001, CFI = 0.955, TLI = 0.942, RMSEA = .093 RMSEA 95% CI = 0.082-0.108, SRMR = 0.034), albeit RMSEA was a bit on the high side. A slight improvement in the fit was done after correlating few items on the DASS-8 (χ^2^ (DF) = 156.34 (40), p = 0.001, CFI = 0.975, TLI = 0.966, RMSEA = .071 RMSEA 95% CI = 0.060-0.083, SRMR = 0.027). In both models, the items of the UCLALS3 had strong significant loadings (>0.7) on their domain-specific factors, in the absence of error correlation of these items with each other or with the items of the DASS-8 (**Figure 2**).

**Figure 3:**
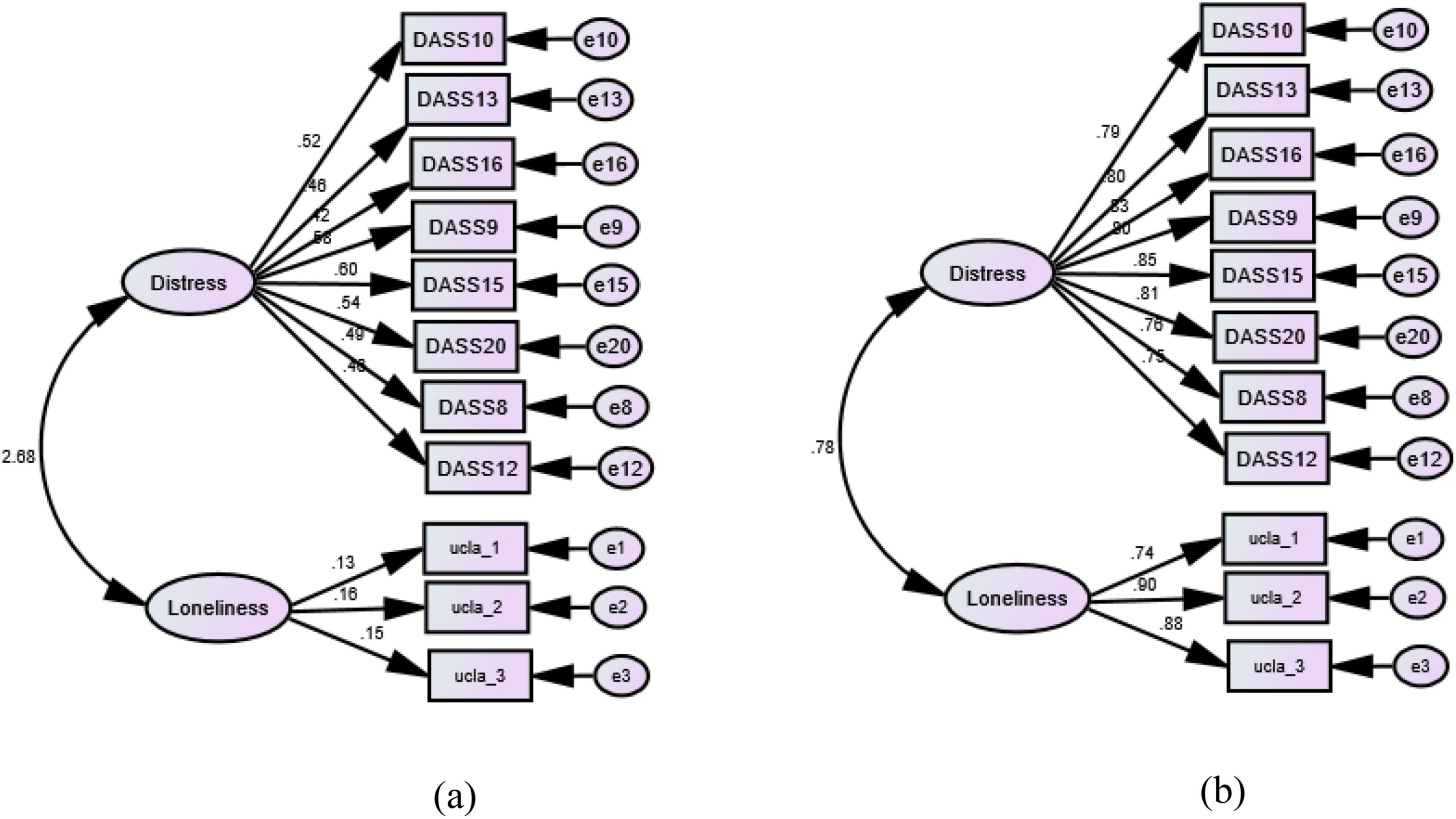
Multigroup confirmatory factor analysis of the Three-Item University of California, Los Angeles, Loneliness Scale-version (UCLALS3) among dementia caregivers across countries. **(a)** Switzerland: χ2 (DF) = 63.03 (43), p = 0.025, CFI = 0.911, TLI = 0.887, RMSEA = .057, SRMR = 0.063. **(b)** Italy: χ2 (DF) = 276.76 (43), p = 0.001, CFI = 0.934, TLI = 0.916, RMSEA = 0.113, SRMR = .041

Multigroup CFA analysis revealed invariance of the UCLALS3 at the configural, metric, scalar, and strict levels across gender. Although the UCLALS3 demonstrated configural invariance across countries, it did not hold scalar invariance (**Table 2**). Accordingly, we have examined the UCLALS3 in each group separately. As shown in **Figure 3**, the fit of the crude model did not vary much from the fit noticed in the whole sample. However, all item loadings (DASS-8 and UCLALS3) decreased in the Swiss sample, and UCLALS3 items expressed an obvious reduction in their loadings, albeit they loaded significantly (p values <0.01). The correlation between the DASS-8 and UCLALS3 in this sample was out of the logical range. Strangely, the fit in this sample was corrected by correlating the error terms of item 3 on the UCLALS3 with item 8 on the DASS-8, which was associated with a greater increase in the association between the DASS-8 and the UCLALS3 (**Supplementary Figure 1**).

**Table 2:** Invariance of the Three-Item University of California, Los Angeles, Loneliness Scale-version (UCLALS3) among dementia caregivers (N = 571).

Groups	Invariance levels	χ^2^	DF	*p*	Δχ^2^	ΔDF	*p*(Δχ^2^)	CFI	ΔCFI	TLI	ΔTLI	RMSEA	ΔRMSEA	SRMR
Gender	Configural Metric Scalar Strict	310.575 319.179 328.902 339.155	86 95 98 109	0.001 0.001 0.001 0.001	8.61 9.72 10.25	9 3 11	0.475 0.021 0.508	.951 .951 .950 .950	0.000 0.001 0.000	.937 .943 .943 .949	-0.006 0.000 -0.006	.068 .064 .064 .061	0.004 0.000 0.003	0.044 0.049 0.082 0.081
Country	Configural Metric Scalar Strict	339.683 340.827 552.548 581.941	86 95 98 109	0.001 0.001 0.001 0.001	1.14 211.72 29.39	9 3 11	0.999 0.001 0.002	.933 .935 .880 .875	-0.002 0.055 0.005	.914 .925 .865 .874	0.011 0.060 -0.009	.072 .067 .090 .087	0.005 -0.023 0.003	0.041 0.041 0.064 0.060

*χ* ^2^, chi-square; DF, degrees of freedom; CFI, comparative fit index; TLI, Tucker–Lewis index; RMSEA, root mean square error of approximation; CI, confidence interval; SRMR, standardized root mean residual; F, factor, values in boldface indicate partial non-variance.

Strong correlations of the UCLALS3 with the ZBI and the DASS-8 (**Table 3**) signify its usefulness as a criterion variable in distressed and burdened groups. The internal consistency of the scale was very good (alpha = 0.87). Moreover, the reduction in the UCLALS3’s reliability upon item deletion (**Table 1**) indicates a strong contribution of each item to the latent structure covered by this measure. High values of item-total correlations reflect adequate convergent validity.

**Table 3:** Criterion validity of the Three-Item University of California, Los Angeles, Loneliness Scale-version (UCLALS3) among dementia caregivers (N = 571).

	1.	2.	3.
1. UCLALS3	–		
2. ZBI	0.569^**^	–	
3. DASS-8	0.736^**^	0.727^**^	–
Alpha coefficient	0.87	0.94	0.93
Mean (SD)	6.8 ± 2.1	13.6 ± 6.9	54.3 ± 18.3

UCLALS3, Three-Item University of California, Los Angeles, Loneliness Scale-version, ZBI, Zarit Burden Interview, DASS-8, Depression Anxiety Stress Scale-8, SD, standard deviation, ** Correlation is significant at the 0.01 level (2-tailed).

Based on percentile scores, five cutoff scores were suggested for the UCLALS3. They may reflect various levels of loneliness (**Table 4**).

**Table 4:** Percentile distribution of the Three-item University of California, Los Angeles, Loneliness Scale-version (UCLALS3) scores among dementia caregivers (N = 571).

	Percentiles	UCLALS3 scores	No (%)
Low	10^th^	3.0	63 (11.0)
Mild	20^th^ 25^th^	5.0 5.0	95 (16.6)
Moderate	30^th^ 40^th^	6.0 6.0	74 (13.0)
High	50^th^ 60^th^ 70^th^	7.0 8.0 8.0	183 (32.0)
Severe	75^th^ 80^th^ 90^th^	9.0 9.0 9.0	156 (27.3)

**Figure 4:**
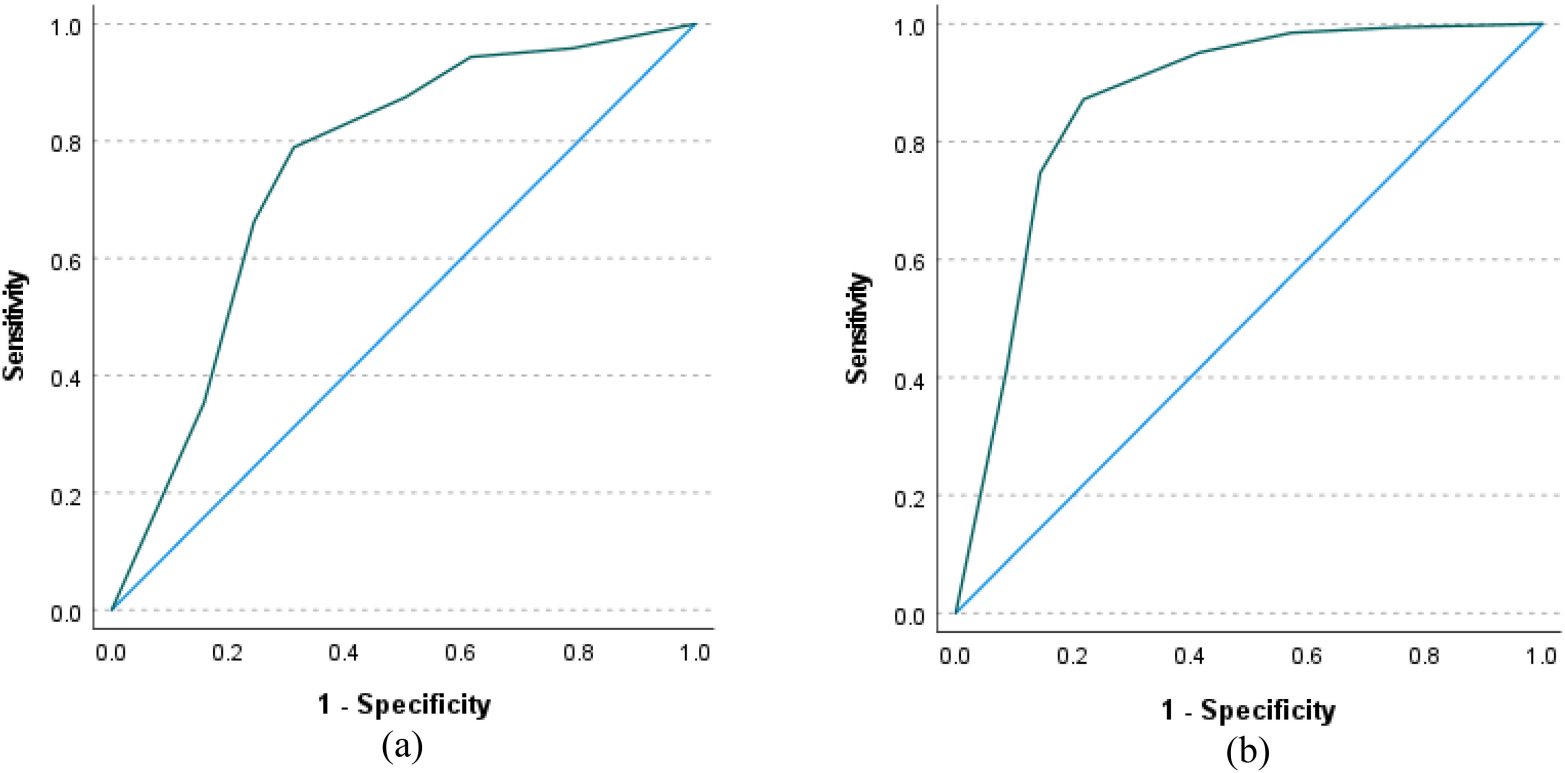
Receiver-operating characteristic (ROC) curve using scores of the Three-Item University of California, Los Angeles, Loneliness Scale-version (UCLALS3) to classify dementia family caregivers according to caregiving burden **(a)** and their level of distress **(b)**.

Most participants (59.3%) had high to severe loneliness (UCLALS3 scores greater than 6). An optimal cutoff score of 6.5 on the UCLALS3 was indicated by the ROC-curve criteria prediction of ZBI and DASS-8 scores (AUC = 0.75 and 0.87, SE = 0.02, p values <0.001, 95% CI: 0.71 to 0.80 and 0.84 to 0.90, sensitivity = 0.79 and 0.87, specificity = 0.69 and 0.78, Youden index = 0.48 and 0.65, respectively) (**Figure 4**). Based on the cutoff score obtained from ROC analysis, 232 (40.6%) respondents had UCLALS3 scores below and equal to 6.5, and 339 (59.4%) had scores greater than 6.5.

The Kruskal-Walli’s test revealed significant differences in caregiving burden and psychological distress across percentile categories (H (4) = 191.29 and 313.40, respectively, p values <0.001), with the highest levels reported in participants with a UCLALS3 score of 7 and above. Likewise, the Mann-Whitney U test revealed significant differences in caregiving burden and distress between low- and high-loneliness groups as defined by the cutoff score produced by the ROC analysis (U = 13318.5 and 6996.0, z = -13.43 and -16.72, p values <0.001).

## 4 Discussion

Poor physical health, psychological distress, and emotional negativity are documented correlates of loneliness, which is evident in a considerable proportion of dementia caregivers (1, 4, 28). Aiming to improve the detection of loneliness in this population, this study examined the psychometric characteristics and the cutoff score of the UCLALS3, the briefest version of the UCLALS, in a sample of Italian and Swiss family caregivers. Our findings indicate the usefulness of the UCLALS3 as a unidimensional measure of loneliness, with an optimal cutoff score of 6.5 for detecting noxious psychological experiences such as caregiving burden and emotional distress.

Limited studies have examined the psychometrics of the UCLALS3. In our study, EFA and CFA showed strong loadings of all the items on a single factor, which explained most of the variance. The scale expressed perfect invariance across gender and invariance only at the configural level across countries. Similarly, the UCLALS3 expressed a good fit for a single factor in the general population and older adults from the USA (7, 50), German older adults (50), Italian and Spanish samples of healthy adults (42, 51), Portuguese community-dwelling adults (52), as well as Japanese mothers of little children (2). The structure of the scale in these studies was primarily examined by EFA or principal component analysis; one factor explained above 70% of the variance (51, 52). All these results support the construct validity of the unifactorial UCLALS3. This finding supports its usability for quick screening for loneliness in epidemiological surveys, given that the literature reports extensive misfit and non-invariance of the UCLALS and most of its shorter versions, with the shortest versions expressing the least non-invariance and highest reliability (33).

Although invariance of the UCLALS3 across gender ensures rigorous comparisons of the levels of loneliness between men and women, more attention should be paid to comparing loneliness at the global level when the UCLALS3 is used due to variance at the metric level across countries, with the Swiss sample displaying extremely low item loadings (<0.2), extraordinary correlation between the DASS-8 and the UCLALS3, as well as correlations of the error terms of item 8 of the former “I felt that I was using a lot of nervous energy” with item 3 of the latter “How often do you feel isolated from others” (**Supplementary Figure 1**). It seems that studies on the invariance of various UCLA scales across countries are quite lacking. However, we located a single study examining invariance of the UCLA-20 and the UCLA-8 across three countries: USA, Germany, and Indonesia. In that study, both measures failed to hold invariance at all levels except for the UCLA-8, which was invariant only at the configural, and the authors had to omit two items to improve the fit of the UCLA-8. Interestingly, the items removed were exclusively related to social isolation—same as item 3 in our study (53). In a Chinese evaluation of the UCLA-8 in older adults through CFA and Rasch analysis, the scale was not unidimensional, item 3 was misfitting, and the response categories were combined. The level of loneliness determined in that study was much lower than those generally reported in that population (54). Moreover, an evaluation of the UCLALS3 in 337 young adults from South Africa suggested that the scale is best conceptualized as comprising three subscales (isolation, relational connectedness, collective connectedness), in addition to the total scale score (55). These findings indicate that despite of the general perception of loneliness, individuals from different countries may vary on how they conceptualize their loneliness experience. Further investigations of this phenomenon across many countries are warranted.

Despite its small number of items, the reliability of the UCLALS3 noticed in our study is high: similar to or greater than that reported in other studies (alpha = 0.89) (2) (omega = 0.78 & alpha = 0.72) (3, 7, 56). The dataset used in our analysis did not comprise the UCLALS or longer shortened versions, which may be used for known tests of convergent/predictive validity. However, the scale displayed adequate convergent validity through strong correlations of all the items with each other and with the total score of the scale (**Table 1**), same as the original UCLALS3 (7). The UCLALS3 also expressed adequate convergent validity in former studies; it had strong positive correlations with the UCLALS10 (2) and the UCLALS20 (2, 7, 33). These results show that the affiliative aspects covered by the UCLALS3 (isolation, relational connectedness, and trait loneliness) are strongly related in a pattern that may reliably reflect significant loneliness.

In the exploration of its criterion validity, we found strong positive correlations of the single factor covered by the UCLALS3 with caregiving burden and distress scores. Similarly, previous studies express strong correlations of the UCLALS3 with depressive symptoms (e.g., feeling lonely, couldn’t get going, and not enjoying life) (7), measures of self-esteem (3), childcare burden, and limited social networks (2). Accordingly, the UCLALS3 may be a useful criterion variable that may indirectly flag numerous negative emotional experiences in distressed groups (e.g., caregivers of chronic conditions, mothers of little children, and older adults).

We used scale percentile scores to distinguish those with high- from low-loneliness at a score above 6. ROC analysis examining the cutoff score of the UCLALS3 in relevance to reported cutoff points of the ZBI and the DASS-8 indicated that a UCLALS3 score of 6.5 and above may effectively reflect caregivers with higher levels of caregiving burden and emotional distress—this score is relatively closer to the suggested percentile score. AUC values in both models (0.75 & 0.87) show that the discriminative potential of the UCLALS3 ranges from good to very good. Furthermore, we examined the differences in caregiving burden and psychological distress across groups defined by cutoff scores produced in both methods. The scores of caregiving burden and psychological distress were significantly greater among those scoring 6 and above, which lends further credibility to the defined cutoff scores. Interestingly, the levels of discrimination between high and low loneliness were greater when a single optimal cutoff was used compared with multiple cutoff scores, which describe different levels of loneliness (low, mild, moderate, high, and severe: **Table 4**). Therefore, a UCLALS3 score of 6.5 may be reliably used to discriminate caregivers with high caregiving burden and emotional distress, who may be subjected to further screening for mental morbidities and be targeted by interventional strategies.

Reports on the prevalence of loneliness among dementia caregivers are scarce. As reported above, loneliness levels observed in the present study (moderate and high) are, somewhat, comparable to those reported in other countries. For example, moderate and severe loneliness are reported in 43.7 and 17.7% of 1283 dementia family caregivers in the United Kingdom (4). However, more participants in our study expressed severe loneliness. This finding can be interpreted with reference to the time of data collection, 2020 when the COVID-19 pandemic was peaking in all countries of the world. Lockdown measures, along with the related unavailability of an alternative caregiver may be possible reasons (14, 15). Indeed, lonely people feel unsafe likely due to activation of an anachronistic survival mechanism that intensifies sensitivity to different threats, including minor ones (32). Other factors may include variations in the loneliness measures used in those studies as well as cultural orientations in different countries—individuals from individualistic cultures (such as the current sample) experience greater levels of loneliness (57).

This study has the advantage of employing already available data to examine the psychometrics of the UCLALS3 and the prevalence of loneliness among dementia caregivers. To our knowledge, this study is the first to statistically determine a cutoff score of the UCLALS3, and this cutoff score seems to efficiently identify individuals with high burnout and distress. Given that only three items could statistically predict caregiving burden and distress, the UCLALS3 may be a cost-effective measure, which may indirectly detect other mental problems in distressed individuals. This may have implications for assessing loneliness-related mental consequences in research and practice. This study; however, comes with many limitations: 1) risk for selection bias because of convenience sampling and online surveying, possibly targeting those with special internet use skills and tendencies, 2) females constituted most of the participants, 3) the status of being a dementia caregiver, as well as the duration of caregiving, and the type of dementia were self-reported, 4) cross-sectional data do not allow a chance to evaluate the stability of symptoms over time, 5) data are limited to a specific geographical area and time period during the peak of the COVID-19 pandemic, 6) it was not possible to assess the concurrent validity of this measure in relevance to commonly used measures of loneliness (the gold standard), and 7) the number of participants in Swiss group was relatively small, which may influence the results (58). Replication of the study may be necessary to ensure the suitability of the scale to various cultural contexts as well as the credibility of the determined cutoff score.

## 5 Conclusions

The findings of the present study provide further evidence for the single-factor structure of the UCLALS3, as well as its measurement invariance across gender, adequate internal consistency, convergent validity, and criterion validity. The scale at a cutoff score of 6.5 may efficiently identify clinical targets: lonely people with considerable levels of burnout and mental distress. According to this optimal cutoff, the prevalence of clinically relevant loneliness in this sample was high (59.4%). The results note that loneliness may at these scores be a likely aspect of intervention to reduce caregivers’ mental suffering. Scale invariance across Swiss and Italian caregivers observed in our study calls for future studies that evaluate invariance of the UCLALS3 across different countries.

## Data Availability

Publicly available datasets were analyzed in this study. This data can be found in the Zenodo open repository: https://zenodo.org/records/4748652.

## References

[1] Achterbergh L Pitman A Birken M Pearce E Sno H Johnson S. The experience of loneliness among young people with depression: a qualitative meta-synthesis of the literature. BMC Psychiatry. (2020) 20:415. doi: 10.1186/s12888-020-02818-3 32831064 PMC7444250

[2] Arimoto A Tadaka E. Reliability and validity of Japanese versions of the UCLA loneliness scale version 3 for use among mothers with infants and toddlers: a cross-sectional study. BMC Womens Health. (2019) 19:105. doi: 10.1186/s12905-019-0792-4 31349835 PMC6660924

[3] Mund M Maes M Drewke PM Gutzeit A Jaki I Qualter P. Would the real loneliness please stand up? The validity of loneliness scores and the reliability of single-item scores. Assessment. (2022) (4):1226–48. doi: 10.1177/10731911221077227 PMC10149889 35246009

[4] Victor CR Rippon I Quinn C Nelis SM Martyr A Hart N. The prevalence and predictors of loneliness in caregivers of people with dementia: findings from the IDEAL programme. Aging Ment Health. (2021) 25:1232–8. doi: 10.1080/13607863.2020.1753014 32306759

[5] Shah SGS Nogueras D van Woerden HC Kiparoglou V. Evaluation of the effectiveness of digital technology interventions to reduce loneliness in older adults: systematic review and meta-analysis. J Med Internet Res. (2021) 23:e24712. doi: 10.2196/24712 34085942 PMC8214187

[6] Surkalim DL Luo M Eres R Gebel K van Buskirk J Bauman A. The prevalence of loneliness across 113 countries: systematic review and meta-analysis. BMJ. (2022) 376:e067068. doi: 10.1136/bmj-2021-067068 35140066 PMC8826180

[7] Hughes ME Waite LJ Hawkley LC Cacioppo JTA. Short scale for measuring loneliness in large surveys: results from two population-based studies. Res Aging. (2004) 26:655–72. doi: 10.1177/0164027504268574 PMC2394670 18504506

[8] Ali AM Al-Dossary SA Fekih-Romdhane F Alameri RA Laranjeira C Khatatbeh H. Psychometric evaluation of the Arabic version of the Eight-Item Center for Epidemiological Studies Depression Scale (CESD-8): Specific cultural considerations for the assessment of depression. Int J Nurs Stud Adv. (2025) 100310. doi: 10.1016/j.ijnsa.2025.100310 PMC12175662 40535791

[9] Ali AM Hori H Kim Y Kunugi H. The Depression Anxiety Stress Scale 8-items expresses robust psychometric properties as an ideal shorter version of the Depression Anxiety Stress Scale 21 among healthy respondents from three continents. Front Psychol. (2022) 13:799769. doi: 10.3389/fpsyg.2022.799769 35496141 PMC9044488

[10] Ali AM Hendawy AO Almarwani AM Alzahrani N Ibrahim N Alkhamees AA. The Six-item Version of the Internet Addiction Test: Its development, psychometric properties, and measurement invariance among women with eating disorders and healthy school and university students. Int J Environ Res Public Health. (2021) 18:12341. doi: 10.3390/ijerph182312341 34886068 PMC8657305

[11] Ali AM Al-Amer R Atout M Ali TS Mansour AMH Khatatbeh H. The Nine-Item Internet Gaming Disorder Scale (IGDS9-SF): Its Psychometric Properties among Sri Lankan Students and Measurement Invariance across Sri Lanka, Turkey, Australia, and the USA. Healthcare. (2022) 10:490. doi: 10.3390/healthcare10030490 35326968 PMC8953588

[12] Kara M Baytemir K Inceman-Kara F. Duration of daily smartphone usage as an antecedent of nomophobia: exploring multiple mediation of loneliness and anxiety. Behav Inf Technol. (2021) 40:85–98. doi: 10.1080/0144929X.2019.1673485

[13] Perez SG Nuccio AG Stripling AMA. Rapid review of the detrimental impact of loneliness and social isolation in caregivers of older adults. Am J Geriatric Psychiatry. (2021) 29:S122–3. doi: 10.1016/j.jagp.2021.01.117

[14] Ali AM Alameri RA Hendawy AO Al-Amer R Shahrour G Ali EM. Psychometric evaluation of the depression anxiety stress scale 8-items (DASS-8)/DASS-12/DASS-21 among family caregivers of patients with dementia. Front Public Health. (2022) 10:1012311. doi: 10.3389/fpubh.2022.1012311 36388286 PMC9641276

[15] Ali AM Alkhamees AA Elhay ESA Taha SM Hendawy AO. COVID-19-related psychological trauma and psychological distress among community-dwelling psychiatric patients: people struck by depression and sleep disorders endure the greatest burden. Front Public Health. (2022) (9):799812. doi: 10.3389/fpubh.2021.799812 35071173 PMC8777039

[16] Khatatbeh H Ali AM Amer F Hammoud S Kurnianto A Boncz I. EPH107 predictors and consequences of pediatric nurses’ Burnout: an integrative review. Value Health. (2023) 26:S222–3. doi: 10.1016/j.jval.2023.09.1149

[17] Alheneidi H AlSumait L AlSumait D Smith AP. Loneliness and problematic internet use during COVID-19 lock-down. Behav Sci. (2021) 11:5. doi: 10.3390/bs11010005 33418914 PMC7825032

[18] Deutrom J Katos V Al-Mourad MB Ali R. The relationships between gender, life satisfaction, loneliness and problematic internet use during COVID-19: does the lockdown matter? Int J Environ Res Public Health. (2022) 19:1325. doi: 10.3390/ijerph19031325 35162348 PMC8835331

[19] Baenas I Caravaca-Sanz E Granero R Sánchez I Riesco N Testa G. COVID-19 and eating disorders during confinement: Analysis of factors associated with resilience and aggravation of symptoms. Eur Eating Disord Rev. (2020) 28:855–63. doi: 10.1002/erv.2771 PMC7461472 32815293

[20] Griffiths MD Kuss DJ Demetrovics Z. Chapter 6 - social networking addiction: an overview of preliminary findings. In: Rosenberg KP Feder LC, editors. Behavioral Addictions. Academic Press, San Diego (2014). p. 119–41.

[21] Ali AM Hori H Kim Y Kunugi H. Predictors of nutritional status, depression, internet addiction, Facebook addiction, and tobacco smoking among women with eating disorders in Spain. Front Psychiatry. (2021) 12:735109. doi: 10.3389/fpsyt.2021.735109 34899416 PMC8663168

[22] Beeson RA. Loneliness and depression in spousal caregivers of those with Alzheimer’s disease versus non-caregiving spouses. Arch Psychiatr Nurs. (2003) 17:135–43. doi: 10.1016/s0883-9417(03)00057-8 12840806

[23] Jaremka LM Andridge RR Fagundes CP Alfano CM Povoski SP Lipari AM. Pain, depression, and fatigue: loneliness as a longitudinal risk factor. Health Psychol. (2014) 33:948–57. doi: 10.1037/a0034012 PMC3992976 23957903

[24] Beeson R Horton-Deutsch S Farran C Neundorfer M. Loneliness and depression in caregivers of persons with Alzheimer’s disease or related disorders. Issues Ment Health Nurs. (2000) 21:779–806. doi: 10.1080/016128400750044279 11854982

[25] Clare L Wu YT Quinn C Jones IR Victor CR Nelis SM. A comprehensive model of factors associated with capability to “Live well” for family caregivers of people living with mild-to-moderate dementia: findings from the IDEAL study. Alzheimer Dis Assoc Disord. (2019) 33:29–35. doi: 10.1097/wad.0000000000000285 30802226 PMC6416095

[26] Peavy G Mayo AM Avalos C Rodriguez A Shifflett B Edland SD. Perceived stress in older dementia caregivers: mediation by loneliness and depression. Am J Alzheimer’s Dis Other Dementias®. (2022) 37:15333175211064756. doi: 10.1177/15333175211064756 PMC10580727 34986661

[27] Ali AM Al-Dossary SA Aljaberi MA Atout M Alamer R Suliman MMH. The Arabic Satisfaction with Life Scale (SWLS) and its three-item version: Factor structure and measurement invariance among university students. Acta Psychol (Amst). (2025) 255:104867. doi: 10.1016/j.actpsy.2025.104867 40073721

[28] Quadt L Esposito G Critchley HD Garfinkel SN. Brain-body interactions underlying the association of loneliness with mental and physical health. Neurosci Biobehav Rev. (2020) 116:283–300. doi: 10.1016/j.neubiorev.2020.06.015 32610178

[29] Maes M Vanhalst J Van den Noortgate W Goossens L. Intimate and relational loneliness in adolescence. J Child Family Stud. (2017) 26:2059–69. doi: 10.1007/s10826-017-0722-8

[30] Cramer KM Barry JE. Conceptualizations and measures of loneliness: a comparison of subscales. Pers Individ Dif. (1999) 27:491–502. doi: 10.1016/S0191-8869(98)00257-8

[31] Shevlin M Murphy S Murphy J. The latent structure of loneliness: testing competing factor models of the UCLA Loneliness Scale in a large adolescent sample. Assessment. (2015) 22:208–15. doi: 10.1177/1073191114542596 25022276

[32] Cacioppo JT Hawkley LC Ernst JM Burleson M Berntson GG Nouriani B. Loneliness within a nomological net: An evolutionary perspective. J Res Pers. (2006) 40:1054–85. doi: 10.1016/j.jrp.2005.11.007

[33] Panayiotou M Badcock JC Lim MH Banissy MJ Qualter P. Measuring loneliness in different age groups: the measurement invariance of the UCLA loneliness scale. Assessment. (2023) 30:1688–715. doi: 10.1177/10731911221119533 PMC10248311 36031881

[34] Messina A Lattanzi M Albanese E Fiordelli M. Caregivers of people with dementia and mental health during COVID-19: findings from a cross-sectional study. BMC Geriatr. (2022) 22:56. doi: 10.1186/s12877-022-02752-x 35034607 PMC8761089

[35] Ali AM Alkhamees AA Hallit S Al-Dwaikat TN Khatatbeh H Al-Dossary SA. The Depression Anxiety Stress Scale 8: investigating its cutoff scores in relevance to loneliness and burnout among dementia family caregivers. Sci Rep. (2024) 14:13075. doi: 10.1038/s41598-024-60127-1 38844485 PMC11156668

[36] Messina A. Caregivers of people with dementia and mental health during COVID-19: findings from a cross-sectional study. Zenodo. (2021). doi: 10.5281/zenodo.4748651 PMC8761089 35034607

[37] Chattat R Cortesi V Izzicupo F Del Re ML Sgarbi C Fabbo A. The Italian version of the Zarit Burden interview: a validation study. Int Psychogeriatr. (2011) 23:797–805. doi: 10.1017/s1041610210002218 21205379

[38] Khatatbeh H Al-Dwaikat T Alfatafta H Ali AM Pakai A. Burnout, quality of life and perceived patient adverse events among paediatric nurses during the COVID-19 pandemic. J Clin Nurs. (2022) 32(13-14):3874–86. doi: 10.1111/jocn.16540 PMC9538583 36123307

[39] Bédard M Molloy DW Squire L Dubois S Lever JA O’Donnell M. The Zarit Burden Interview: a new short version and screening version. Gerontologist. (2001) 41:652–7. doi: 10.1093/geront/41.5.652 11574710

[40] Bottesi G Ghisi M Altoè G Conforti E Melli G Sica C. The Italian version of the Depression Anxiety Stress Scales-21: Factor structure and psychometric properties on community and clinical samples. Compr Psychiatry. (2015) 60:170–81. doi: 10.1016/j.comppsych.2015.04.005 25933937

[41] Ali AM Alkhamees AA Hori H Kim Y Kunugi H. The depression anxiety stress scale 21: development and validation of the depression anxiety stress scale 8-item in psychiatric patients and the general public for easier mental health measurement in a post-COVID-19 world. Int J Environ Res Public Health. (2021) 18:10142. doi: 10.3390/ijerph181910142 34639443 PMC8507889

[42] Bottaro R Valenti GD Faraci P. Assessment of an epidemic urgency: psychometric evidence for the UCLA loneliness scale. Psychol Res Behav Manag. (2023) 16:2843–55. doi: 10.2147/PRBM.S406523 PMC10387257 37525851

[43] Noureddine A Malaeb D El Khatib S Dabbous M Sakr F Ali AM. Psychometric properties of an Arabic translation of the 13-item short mood and feelings questionnaire- parent version (SMFQ-P) to screen for depression in children. BMC Psychiatry. (2025) 25:2. doi: 10.1186/s12888-024-06433-4 39748341 PMC11697484

[44] Ali AM Al-Dossary SA Laranjeira C Amer F Hallit S Alkhamees AA. Effects of hormonal replacement therapy and mindfulness-based stress reduction on climacteric symptoms following risk-reducing salpingo-oophorectomy. Healthcare. (2024) 12:1612. doi: 10.3390/healthcare12161612 39201170 PMC11353799

[45] Khatatbeh H Amer F Ali AM M AL Kurnianto A Abu-Abbas M. Challenges of distance learning encountering nursing students after the COVID-19 pandemic: a study from the Middle East. BMC Nurs. (2024) 23:574. doi: 10.1186/s12912-024-02236-w 39154168 PMC11330021

[46] Yu Y Liu Z-W Zhou W Zhao M Qiu D Li Y-L. Cutoff of the Zarit Burden Interview in predicting depression and anxiety. Qual Life Res. (2019) 28:2525–33. doi: 10.1007/s11136-019-02208-7 31089989

[47] Ali AM Alameri RA Brooks T Ali TS Ibrahim N Khatatbeh H. Cut-off scores of the Depression Anxiety Stress Scale-8: Implications for improving the management of chronic pain. J Clin Nurs. (2023) 32(23-24):8054–62. doi: 10.1111/jocn.16878 37674274

[48] Ali AM Al-Dossary SA Almarwani AM Atout M Al-Amer R Alkhamees AA. The Impact of Event Scale-Revised: Examining Its Cutoff Scores among Arab Psychiatric Patients and Healthy Adults within the Context of COVID-19 as a Collective Traumatic Event. Healthcare. (2023) 11:892. doi: 10.3390/healthcare11060892 36981549 PMC10048280

[49] Ali AM Al-Dossary SA Laranjeira C Atout M Khatatbeh H Selim A. Cardiometabolic morbidity (Obesity and hypertension) in PTSD: A preliminary investigation of the validity of two structures of the impact of event scale-revised. J Clin Med. (2024) 13:6045. doi: 10.3390/jcm13206045 39457995 PMC11509123

[50] Hawkley LC Duvoisin R Ackva J Murdoch JC Luhmann M. Loneliness in older adults in the USA and Germany: Measurement invariance and validation. Working Paper Series, NORC at the University of Chicago, Paper 2015–002 (2015).

[51] Trucharte A Calderón L Cerezo E Contreras A Peinado V Valiente C. Three-item loneliness scale: psychometric properties and normative data of the Spanish version. Curr Psychol. (2023) 42:7466–74. doi: 10.1007/s12144-021-02110-x PMC8285042 34305365

[52] Daniel F Espírito-Santo H Lemos L Guadalupe S Barroso I da Silva AG. Measuring loneliness: Psychometric properties of the three-item loneliness scale among community-dwelling adults. Heliyon. (2023) 9:e15948. doi: 10.1016/j.heliyon.2023.e15948 37215896 PMC10192737

[53] Hudiyana J Lincoln TM Hartanto S Shadiqi MA Milla MN Muluk H. How universal is a construct of loneliness? Measurement invariance of the UCLA loneliness scale in Indonesia, Germany, and the United States. Assessment. (2022) 29:1795–805. doi: 10.1177/10731911211034564 34301150

[54] Zhong Q Jiang Y Conwell Y Chen S. Can the short-form UCLA loneliness scale be used to measure loneliness among Chinese older adults? From classical test theory to rasch analysis. Int J Geriatr Psychiatry. (2024) 39:e70017. doi: 10.1002/gps.70017 39648272

[55] Laher S. Looking inward: Reflections on the African Journal of Psychological Assessment and the way forward. Afr J psychol Assess. (2022) 4:3. doi: 10.4102/ajopa.v4i0.132

[56] Roberts RE Lewinsohn PM Seeley JR. A brief measure of loneliness suitable for use with adolescents. Psychol Rep. (1993) 72:1379–91. doi: 10.2466/pr0.1993.72.3c.1379 8337350

[57] Barreto M Victor C Hammond C Eccles A Richins MT Qualter P. Loneliness around the world: Age, gender, and cultural differences in loneliness. Pers Individ Dif. (2021) 169:110066. doi: 10.1016/j.paid.2020.110066 33536694 PMC7768187

[58] Ali AM Hendawy AO Al-Amer R Shahrour G Ali EM Alkhamees AA. Psychometric evaluation of the Depression Anxiety Stress Scale 8 among women with chronic non-cancer pelvic pain. Sci Rep. (2022) 12:20693. doi: 10.1038/s41598-022-15005-z 36450770 PMC9712382

