# Supplementary figures and images for: Loneliness among dementia caregivers: evaluation of the psychometric properties and cutoff score of the Three-item UCLA Loneliness Scale
Source: Front Psychiatry. 2025 Apr 7;16:1526569. doi: 10.3389/fpsyt.2025.1526569 (PMC12010107; doi:10.3389/fpsyt.2025.1526569)

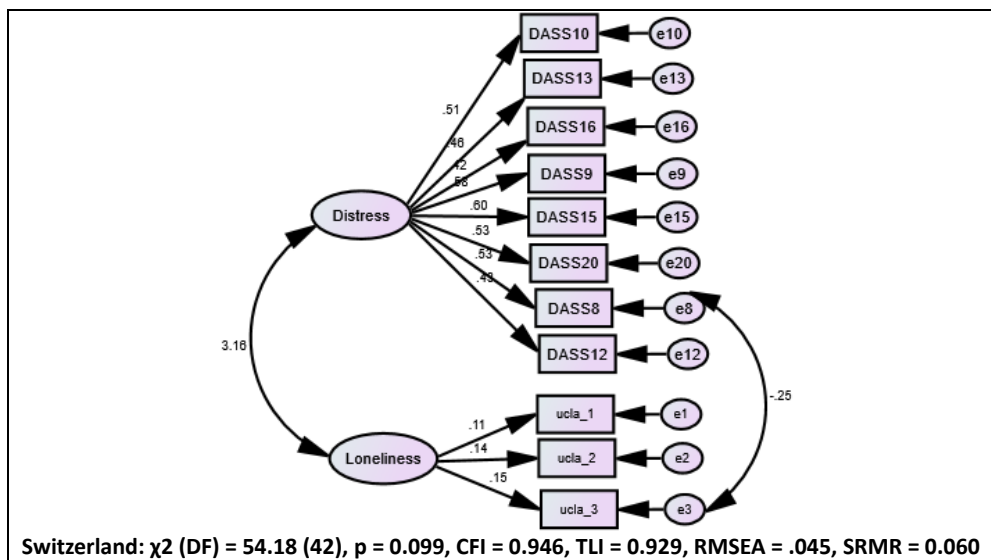

Supplementary Figure 1. Modified CFA of the UCLAALS3 and fit indices in the Swiss group.

Supplement: Supplementary file 1 [file Image1.pdf]
